# Protective and risk factors associated with substance use coping among healthcare workers during the COVID-19 pandemic

**DOI:** 10.3389/fpsyg.2023.1228517

**Published:** 2023-12-20

**Authors:** Vaughn E. Bryant, Michael J. Sorna, Audrey Dana, Kalie G. Leon, Andrea D. Guastello, Nicola Sambuco, Ashley Huxhold, Brandon Allen, Steven P. Cuffe, Carol A. Mathews, Lourdes P. Dale

**Affiliations:** ^1^Department of Psychiatry, College of Medicine – Jacksonville, University of Florida, Jacksonville, FL, United States; ^2^Department of Psychology, University of North Florida, Jacksonville, FL, United States; ^3^Department of Psychiatry, College of Medicine, University of Florida, Gainesville, FL, United States; ^4^Department of Clinical and Health Psychology, College of Public Health and Health Professions, University of Florida, Gainesville, FL, United States; ^5^Department of Emergency Medicine, College of Medicine, University of Florida, Gainesville, FL, United States; ^6^Department of Psychiatry, Center for OCD, Anxiety and Related Disorders, College of Medicine, University of Florida, Gainesville, FL, United States

**Keywords:** substance use coping, healthcare workers, COVID-19, burnout, moral injury, anxiety, interpersonal disengagement, professional fulfillment substance use coping

## Abstract

**Background:**

Healthcare workers (HCWs) experienced high levels of stress and mental health consequences associated with the COVID-19 pandemic, which may have contributed to unhealthy coping behaviors, such as substance use coping (SUC). This study aimed to understand the extent of and predictors of SUC.

**Methods:**

The sample consisted of 263 HCWs in North Central Florida. Univariable and multivariable logistic regression analyses investigated whether moral injury and other work risk factors, protective factors, and clinically relevant symptoms (i.e., work exhaustion, interpersonal disengagement, depression, anxiety, and/or PTSD) were associated with likelihood of SUC.

**Results:**

Clinically relevant levels of interpersonal disengagement and anxiety increased the likelihood of SUC. Mediational analyses found that interpersonal disengagement and anxiety explained 54.3% of the relationship between Self Moral Injury and SUC and explained 80.4% of the relationship between professional fulfillment and SUC.

**Conclusion:**

Healthcare supervisors should be aware that providers who are experiencing moral injury and less professional fulfillment may be experiencing significant interpersonal disengagement and anxiety, which could lead to SUC. Future studies should examine the effects of implementing targeted prevention and treatment interventions, along with longitudinal outcomes related to SUC behaviors.

## Introduction

For decades, there has been an interest in examining how stress in the work environment relates to job performance, workload, patient care, and mental health outcomes among healthcare workers (HCWs). Research on HCWs suggests that workplace stressors (e.g., inadequate staffing, high patient-to-provider ratios, excessive workloads, time constraints, and coping with patient death) are associated with increased rates of burnout (Norman et al., 2021) and two times higher rates of anxiety and depression than the rates in the general population (Calnan et al., 2001; Mark and Smith, 2012).

The onset of the COVID-19 pandemic further increased the levels of stress and emotional exhaustion among HCWs as they faced, among other stressors, high patient mortality rates, professional task saturation, and limited access to personal protective equipment (Ahmed et al., 2021; Foli et al., 2021a,b). For example, rates of emotional exhaustion among HCW increased from 31% in 2019 (pre-pandemic) to 40% in 2022 (mid-pandemic) (Sexton et al., 2022). The effects of prolonged periods of stress on HCWs’ wellbeing during the pandemic has been compared to combat stress given the potentially traumatic work environments, particularly in emergency departments (Cipolletta and Ortu, 2021). Research suggests that HCWs in COVID-19 units were exposed to more patient deaths and were more likely to report posttraumatic stress symptoms than those in other units (Mosheva et al., 2021). In addition, HCWs have faced, at increased rates, the possibility of getting infected with the SARS-CoV2 virus, the risk of spreading the virus to loved ones, limited access to personal protective equipment, and a decreased ability to provide adequate patient care (Halcomb et al., 2020; García-Martín et al., 2021). In particular, HCWs with insufficient resources experienced higher levels of interpersonal disengagement from patients and emotional distress, both of which increased the risk of decreased job performance and decreased quality of patient care (Dyrbye et al., 2019; Kakemam et al., 2021). Elevated levels of the three core dimensions of burnout- emotional exhaustion, depersonalization/interpersonal disengagement, and reduced sense of professional accomplishment- have been linked to an increase in errors made by HCWs and a perceived poor quality of patient care (Poghosyan et al., 2010; Van Bogaert et al., 2010, 2013, 2014; Hayashino et al., 2012; Nantsupawat et al., 2016; Sulaiman et al., 2017; Trockel et al., 2018; Tawfik et al., 2019; Kakemam et al., 2021). COVID-19 related work stressors have led to negative mental health effects in addition to increased risk of burnout, including anxiety, depression, emotional distress (Foli et al., 2021a,b; Galanis et al., 2021; Manzano García and Ayala Calvo, 2021; Sarabia-Cobo et al., 2021), and suicide (Kingston, 2020). As a result, there is now legislation focused on improving mental and behavioral health among HCWs (Public Law No: 117–105; 03/18/2022).

Chronic stress is a well-known risk factor for substance use and misuse, the development of substance use disorder, and relapse of a substance use disorder (Sinha, 2008; Al’absi, 2018; Ruisoto and Contador, 2019). Independent of the effects of the COVID-19 pandemic, research suggests that HCWs misuse prescription substances at an elevated rate and use illicit substances at a rate similar to that of the general population (Hughes et al., 1992; Dumitrascu et al., 2014). Less is known about the rates of substance use coping (SUC), in part because individuals may under-report substance use out of the desire to self-preserve or fear of legal or regulatory repercussions (Graham et al., 2001; Weaver et al., 2001; Dumitrascu et al., 2014). Although there are reports of increases in substance use as a means to cope with COVID-19 stressors in the general population (Czeisler et al., 2020; Panagiotidis et al., 2020; Taylor et al., 2021), little research has examined this trend in HCWs. However, a qualitative study found that nurses reported using more substances such as alcohol, marijuana, and tobacco as a coping behavior and openly discussed their increased use with one another (Foli et al., 2021a, 2021b). In addition, excessive substance use and/or misuse is associated with burnout and poorer mental health, including increased symptoms of anxiety and depression (Faltz, 1998; Oreskovich et al., 2015; McCain et al., 2017; De Junqueira et al., 2018; Patel et al., 2019; Foli et al., 2021a,b; Ziarko et al., 2022). Several protective factors have been associated with lower rates of substance use and misuse, including strong support systems, spirituality, positive social engagement, resiliency, good problem-solving skills, self-confidence, and level of education (Family and Social Services Administration, 2020).

The relationship of substance use coping and moral injury among HCWs is also unknown. Moral injury (MI) is defined as the perpetration, failure to prevent, or witnessing of an event that violates the provider’s own moral code, resulting in long-term emotional, psychological, biological, spiritual, and/or social consequences (Litz et al., 2009). In the context of the COVID-19 pandemic, research has focused on rates and correlates of MI in HCWs because they may experience a moral dilemma in the context of trying to provide the best patient care while simultaneously having to make potentially life-or-death decisions with limited resources (Kröger, 2020). Using the same dataset as the current study, Dale et al. (2021) found that HCWs experienced consistently high rates of MI, and that Self MI (i.e., acting against one’s own morals or failing to engage in an action consistent with one’s morals and feeling troubled by it) and Others MI (i.e., seeing something inconsistent with one’s morals and feeling troubled by it) were differentially associated with specific risk factors and outcomes. For example, Others MI (but not Self MI) was associated with predisposing factors such as prior mental health adversity, while Self MI was associated with greater symptoms of depression, anxiety, PTSD, and professional burnout than was Others MI. Furthermore, the Dale et al. study highlighted the need to independently consider the individual components of burnout (i.e., work exhaustion and interpersonal disengagement), as participants experiencing greater worry about the health consequences of COVID-19 reported higher levels of work exhaustion, and those more impacted by the care they were providing to the COVID-19 patients reported higher levels of interpersonal disengagement. However, while research was useful in explaining the factors that lead to moral injury and the psychiatric difficulties experienced by HCWs, it did not address the coping mechanisms that were being employed to manage these symptoms.

The aim of this study was to estimate the prevalence of SUC among HCWs and explore how SUC may relate to the components of MI (i.e., Self and Others MI) and burnout (i.e., work exhaustion and interpersonal disengagement). We explored the contributions of COVID-19 work stressors (health worry, diagnosis, work impact, and healthcare morally distressing experiences, called HMDEs) and clinically relevant symptoms (i.e., work exhaustion, interpersonal disengagement, depression, anxiety, and PTSD). We also sought to determine whether internal factors, such as personal resilience and professional fulfillment, served as protective factors. In addition, we explored the potential benefits of perceived leadership support, as prior research (e.g., Dale et al., 2021; Norman et al., 2021) suggests that perceived leadership support may mitigate or ameliorate the symptoms of burnout in HCWs. Specifically, we hypothesized the following7:

- COVID-19 stressors, moral injury, and clinically relevant symptoms (i.e., work exhaustion, interpersonal disengagement, depression, anxiety, and PTSD) would be associated with an increased likelihood of SUC.
- Greater personal resilience, professional fulfillment, and perceived leadership support would be associated with a decreased likelihood of SUC.

We also explored whether demographic and personal factors (e.g., age, gender, income, work location) impacted the likelihood of SUC. We explored the potential contributions of healthcare roles (e.g., doctor, nurse, or assistant/technician) because longitudinal research suggests that nurses working during the COVID-19 pandemic reported increased burnout and decreased fulfilment relative to doctors and other HCWs (Guastello et al., 2022). Lastly, we explored the potential effects of being in a committed/marriage-like relationship as prior research suggests that individuals in committed relationships experience less mental distress (Nayak et al., 2021), including less anxiety, depressive, and burnout symptoms (Vanderhorst and McLaren, 2005; Afifi et al., 2006; Meyer and Paul, 2011; Zhou et al., 2022; Meng and Yang, 2023).

## Methods

### Participant recruitment and data collection

The procedures used in this longitudinal study were approved by the Institutional Review Board of the [edited out for blind review]. This study was advertised via flyers distributed in hospitals, nursing homes, and outpatient clinics in two cities in the south of the United States. Prospective participants were eligible to participate if they worked in a healthcare setting in this region, regardless of their type of employment. Although flyers were distributed across multiple locations in two cities, the primary recruitment came from two academic medical centers affiliated with a state university system. One of the centers is a safety net hospital in a large city that receives some funding from the city to care for the indigent population, and the other center is a large tertiary care hospital in a mid-size city. A brochure detailing the study was also emailed to HCWs and other healthcare workers from the department head or administrator at these two academic hospitals. During the data collection, there was a spike in rates of COVID-19 related hospitalization at both primary sites, with the COVID-19 caseloads exceeding capacity in the large city.

Upon enrollment, participants provided informed consent and subsequently completed a core set of assessments at baseline. They were then sent repeat assessments again every month for 7 months for a total of eight possible timepoints (Table 1). Some timepoints (e.g., timepoint 2) included additional optional assessments that were available for completion. In total, there were 209 unique items across all questionnaires that could be completed by participants. Not all items were assessed at every timepoint. Each assessment took between 15 and 20 min to complete. Data were obtained at baseline, 1, 2, 3, 4, 5, 6, and 8 months. Compensation was provided for each of the completed questionnaires. Compensation increased exponentially, concurrent with the number of assessments completed, with the total possible compensation being USD 220 for completion of all possible assessments over the total eight-month period. We also included a table that describes the constructs and measures.

**Table 1:** Constructs, measures, and number of items.

Construct	Measure	Number of items
**Substance use coping**	Carver, 1997	2
**Protective factors**		
Personal resilience	Brief Resilient Coping Scale (Sinclair and Wallston, 2004)	4
Professional fulfillment	Professional fulfillment index (Trockel et al., 2018)	6
Leadership support	Leadership behavior description questionnaire (McDaniel et al., 1973)	14
**Risk factors**		
COVID-19 work stressors		
Health worry	Designed for study	4
Diagnosis	Designed for study	1
Work impact	Designed for study	6
Healthcare morally distressing events	Designed for study	4
Moral injury		
Self moral injury	Moral injury events scale (Nash et al., 2013)	4
Others moral injury	Moral injury events scale (Nash et al., 2013)	2
Clinically relevant symptoms		
Work exhaustion	Professional fulfillment index (Trockel et al., 2018)	4
Interpersonal disengagement	Professional fulfillment index (Trockel et al., 2018)	6
Depression	Patient health questionnaire – 8 (Kroenke et al., 2001)	8
Anxiety	Generalized anxiety disorder – 7 (Spitzer et al., 2006)	7
PTSD	PTSD checklist-5 (Price et al., 2016)	8

Although hospital workers such as patient sitters, clerical and other administrative support staff, and food service workers were eligible for participation in the larger study and included in data collection, they were not included in these analyses. The analyses described in this study focus solely on the baseline data for the participants who had direct patient contact. As reported by this research team in 2021 and presented in Table 1, more than half of the 265 HCWs were nurses, including nurse practitioners. The sample also included some medical assistants and technicians and a large number of doctoral level professionals, who were predominantly medical doctors but also dentists and psychologists. Two of these participants did not complete the substance use coping questions and were not included in this study; therefore, the sample size for these analyses was 263.

### Constructs and measures

Table 1 list the constructs included in the current study. The table also reports the references for each measure and total number of items for each scale.

### Substance use coping

The two-item substance use subscale of the Brief Cope scale (Carver, 1997) was used to assess SUC. The first item is *Using alcohol or other drugs to make myself feel better* and the second item is *I’ve been using alcohol or other drugs to help me get through it.* Both questions are answered via a 4-point Likert scale (0 = *not at all*, 1 = *a little*, 2 = *a medium amount*, 3 = *a large amount*). In the current study, we focused on the internally consistent total score (*α* = 0.90). We also grouped the participants according to whether they endorsed any SUC (responded *a little bit*, *a medium amount,* or *a lot*) on either or both items or denied SUC on both items.

### Protective factors

We focused on personal resilience, professional fulfillment, and perceived leadership support as potential protective factors. To assess personal resilience, the Brief Resilient Coping Scale (Sinclair and Wallston, 2004) was used. This scale consists of four items, *I look for creative ways to alter difficult situations; Regardless of what happens to me, I believe I can control my reaction to it; I believe I can grow in positive ways by dealing with difficult situations;* and *I actively look for ways to replace the losses I encounter in life.* This measure uses a 5-point Likert Scale *(*1 *= does not describe me at all,* 2 *= does not describe me,* 3 *= neutral,* 4 *= describes me, and* 5 *= describes me very well)* for the four internally consistent items (*a* = 0.90). We used the suggested grouping of 4–13 to indicate low resiliency, 14 to 16 to indicate mid resiliency, and 17–20 to indicate high resiliency (Sinclair and Wallston, 2004).

To assess professional fulfillment, the corresponding subscale of the Professional Fulfillment Index (PFI) (Trockel et al., 2018) was used, which asks HCWs how fulfilled they are via a 5-point Likert scale (0 = *not at all true* to 4 = *completely true*) for six items (*a* = 0.90). An example item on the PFI is “During the past 2 weeks my work is satisfying to me.” For this measure, higher scores indicate greater professional fulfillment. For use in some *post hoc* analyses, described further below, we also we devised a “lack of professional fulfillment” score. This was achieved by reverse scoring the items to align them with the overall negative theme. This was done to enhance interpretation and ensure consistency of direction across scales in some analyses.

The Leadership Behavior Description Questionnaire (McDaniel et al., 1973) was used to assess leadership support. This 14-item measure asks participants about their perception of their hospital leadership (participant-defined, from direct supervisor through hospital administration) at making/communicating decisions and incorporating the employee’s input into decision-making, as well as the employee’s sense of belonging and role in the healthcare structure and team, via a 5-point Likert scale (0 = never and 4 = always) for all 14 items (*a* = 0.75).

### Risk factors

We focused on COVID-19 stressors, moral injury, and clinically relevant symptomatology as potential risk factors. With regard to COVID-19 stressors, participants indicated their level of worry that they would be infected with the COVID-19 virus while providing medical care, be infected with the COVID-19 virus in their home or community, become seriously ill because of COVID-19, or infect an immediate family member if they get COVID-19. These COVID-19 health worry questions were answered via a 4-point Likert scale (0 = *not worried* to 3 = *very worried*). The scores for each of the 4 questions were summed to create the COVID-19 Health Worry total score (*a* = 0.85). Participants also indicated whether they had been diagnosed with COVID-19.

Participants also indicated the impact of COVID-19 on their functioning at work, including how impacted they were by their: exposure face-to-face with possible asymptomatic patients, exposure to people under investigation for COVID-19, direct care of patients with COVID-19, performance of procedures (e.g., intubations) in close proximity to patients with COVID-19, care of 1 or more patients who died from COVID-19, and work at the morgue with patients who died from COVID-19. These questions were answered via a 5-point Likert scale (0 = *event did not occur* to 4 = *big impact on my life*) for this 6-item measure (*a* = 0.80).

In addition, participants also responded to four questions that related to their perceived ability or inability to provide optimal care (termed health care quality in Table 1) during the COVID-19 pandemic. Specifically, they were asked whether they were able to conduct necessary assessments or procedures, provide care to patients at the appropriate frequency, refer patients for necessary procedures, and refer patients to specialists. For these items, HCWs who disagreed (e.g., reported being unable to provide appropriate care) were considered to have experienced healthcare moral distress. Total scores were calculated to represent the total number of morally distressing experiences (i.e., HMDEs).

We assessed moral injury via the Moral Injury Events Scale (Nash et al., 2013), which assesses the occurrence of, anguish associated with, and perception of betrayal associated with MI. In the current study, we excluded the questions focused on the perception of betrayal to limit the burden on the participants. Instead, we focused on the six questions assessing level of agreement via a 6-point Likert scale (0 = *strongly disagree* to 5 = *strongly agree*) about the occurrence/anguish of moral injury perpetrated by HCWs themselves and witnessed MI perpetrated by others. As previously reported (Nash et al., 2013), we focused on whether or not participants perceived a transgression of self, which we term Self MI (i.e., acting against one’s own morals or failing to act consistent with morals and feeling troubled by it; 4 items; *α* = 0.94), and perceived betrayal by others, which we termed Others MI (i.e., seeing something that they believed was morally wrong and feeling troubled by it; 2 items; *α* = 0.88).

With regard to current symptoms, we used the work exhaustion and the interpersonal disengagement subscales of the Professional Fulfillment Index (Trockel et al., 2018), which asks HCWs to answer questions related to their attitudes about their work via a 5-point Likert scale (0 = *not at all true* to 4 = *completely true*) to assess these components of burnout. The work exhaustion subscale (4 items; *a* = 0.90), assesses sense of dread, physical/emotional exhaustion, and lack of enthusiasm, and the interpersonal disengagement subscale (6 items; *a* = 0.90) assesses empathy and connection with others, particularly patients and colleagues. To allow for comparisons between the two scales, mean scores were calculated. As suggested in the literature, HCWs who had mean scores 1.33 or higher were considered to be experiencing clinically relevant levels of work exhaustion and/or interpersonal disengagement (Trockel et al., 2018). To determine which aspects of interpersonal disengagement were potentially associated with SUC in post-hoc analyses (i.e., disengagement from colleagues and disengagement from patients), two variables were created from the six items in this scale. Disengagement from patients included three items that measured the same construct (*α* = 0.88) and disengagement from colleagues included two items that measured the same construct (*α* = 0.81).

With respect to current psychiatric symptomatology, the Patient Health Questionnaire - 9 item scale (PHQ-9; Kroenke et al., 2001) was used to measure depressive symptoms (α = 0.88). The Generalized Anxiety Disorder - 7 item scale (GAD-7; Spitzer et al., 2006) was used to measure anxiety symptoms (α = 0.92). The 8 item PTSD Checklist-5 (PCL-5; Price et al., 2016) was used to measure PTSD symptoms (α = 0.90). For both the PHQ-9 and GAD-7, we used the suggested clinical cutoff of 10 or greater (Spitzer et al., 2006), whereas for the PCL-5 we used the suggested clinical cutoff of 19 or greater (Price et al., 2016).

### Statistical analyses

Data were analyzed using IBM SPSS Statistics for Windows, Version 28.0. Armonk, NY: IBM Corp. In addition to descriptive statistics, univariate binary logistic regression analyses were used to determine which demographic characteristics, protective factors (i.e., personal resilience, professional fulfillment, and leadership support), and risk factors (e.g., COVID-19 work stressors, HMDEs, Self and Others MI, clinically relevant psychiatric symptoms) were individually associated with an increased likelihood of SUC. Multivariable forward conditional binary logistic regression analyses (using *p* < 0.05 in the univariable analyses as the inclusion cutoff) were used to identify factors that differentiated between HCWs who reported any SUC and those who denied SUC. Follow-up post-hoc analyses were conducted as relevant to determine whether specific subscales or components of a given measure (e.g., disengagement from patients vs. disengagement from colleagues on the PFI disengagement subscale, individual COVID-19 related items) were associated with an increased likelihood of SUC. Similarly, post-hoc analyses were conducted to determine significant associations between demographic factors and protective factors with identified clinical risk factors for SUC.

Finally, we conducted *post hoc* mediation analyses using SPSS Process model 4 to explore in more depth the relationships between SUC and variables of interest that arose from our primary analyses. As mediation analysis does not allow for the inclusion of categorical variables, we used quantitative scores for these analyses. The hypothesized mediation models were tested using a bootstrapping approach in multiple models to assess the significance of the indirect effects. The PROCESS macro model 4 with bias-corrected 95% confidence intervals (*n* = 10,000) was used to test the whether the indirect (i.e., mediated) effects were mediated by each of the mediators (i.e., conditional indirect effects). Significant effects are indicated by the absence of zero within the confidence intervals. The percent of total effects were calculated for each indirect effect and the remaining direct effect by dividing each coefficient effect by the total effect.

## Results

A total of 263 HCWs were included in the analyses, more than half of whom were nurses (Table 2). Participants varied in age from 20 to 72 years old (*M* = 37.55, *SD* = 11.07), and primarily identified as female and white. The majority had a college education or higher, and reported being in a married/committed relationship. About 40% of the sample reported prior psychiatric treatment, psychotherapy and/or medications. Table 2 reports the percent of HCWs that fell into the low, mid, and high resilient coping groups. No participants received scores indicating high resiliency and the majority of participants received scores indicating low resiliency. Table 2 also reports the percent of HCWs that reported experiencing Self and Others MI and scored above the clinical cutoff with regard to their burnout and psychiatric symptoms.

**Table 2:** Characteristics of healthcare providers (*N* = 263).

Characteristics	*N*	%	Characteristics	*N*	%
**Gender**			**Occupation**		
Female	216	82.4	Doctor	80	33.8
Male	46	17.5	Nurse	128	54.0
**Race**			Medical assistant	29	12.2
White	204	77.6	**Psychiatric treatment history**		
Non-White	59	22.4	Therapy	21	8.0
**Married/committed relationship**			Medication	27	10.3
Yes	166	63.1	Both	57	21.7
No	97	36.9	**Resilient coping**		
**Education**			Low	212	80.9
H.S. Degree	14	5.4	Mid	50	19.1
College Degree	134	51.0	High	0	0.0
Graduate Degree	100	38.0	**Moral injury**		
**Yearly income**			Perpetrated by Self	27	10.3
≤ $40,000	35	13.3	Perpetrated by Others	82	31.2
$40,001 – $60,000	43	16.3	**Scored above clinical cut-off**		
$60,001 – $80,000	44	16.74	Anxiety	66	25.1
$80,001 – $100,000	31	11.8	Depression	64	24.3
$100,001 – $200,000	63	24.0	PTSD	31	11.9
> $200,000	35	13.3	Work exhaustion	166	63.1
**Work location**			Interpersonal disengagement	83	31.6
Large city	161	63.9	**COVID-19 diagnosis**	24	9.2
Small city	91	36.1			

### Substance use coping

With regard to the two items that asked about substance use coping, approximately one third of the participants reported *using alcohol or other drugs to feel better* (20.9% reported a little bit, 10.3% reported a medium amount, and 1.9% reported a lot). A similar percentage reported *using alcohol or other drugs to help me get through it* (19.8% reported *a little bit*, 5.3% reported *a medium amount*, 1.9% reported *a lot*). Because of the likelihood of under-reporting of substance use, individuals who endorsed any SUC on either or both items (*n* = 92, 35.0% of total sample) were place in the substance use coping group and those that denied any SUC on both items (*n* = 171, 65.0% of total sample) were placed in the no substance use group. This binarized group categorization was used as the dependent variable in the univariate and multivariable binary logistic regression analyses. Total scores ranged from 2 (reported *not at all* for both items) to 8 (report *a lot* for both items), with the mean score being 2.83 (*SD* = 1.36).

### Variable impacting likelihood of substance use coping

Table 3 displays the results of the univariable binary regression analyses. Self MI was associated with a significantly increased odds of SUC (OR = 3.06, *p* = 0.007). The odds of SUC were also significantly increased for the HCWs reporting clinically relevant symptoms of interpersonal disengagement (OR = 2.55, *p* < 0.001), depression (OR = 2.53, *p* = 0.002), anxiety (OR = 4.29, *p* < 0.001), and PTSD (OR = 4.70, *p* < 0.001). The only protective factor that was associated with a significantly decreased likelihood of SUC was professional fulfillment (OR = 0.64, *p* = 0.003). As evident in Table 3, work exhaustion, Others MI, and leadership support were not significantly associated with SUC.

**Table 3:** Results of binary logistic regressions predicting likelihood of substance use coping.

**Factors**	**Univariable results**		**Multivariable results**	
	**OR**	**95% CI**	**OR**	**95% CI**
**Demographic factors**				
Age	0.99	0.96–1.01		
Male gender	0.60	0.29–1.23		
white race	1.17	0.63–2.17		
Educational level	0.90	0.61–1.33		
Income	0.96	0.83–1.12		
Married/committed relationship	0.61	0.36–1.02		
Work in large city	1.011	0.59–1.73		
Profession				
Doctor (versus eveyone else)	0.97	0.55–1.72		
Nurse (versus everyone else)	1.42	0.81–2.52		
Medical Assistant (versus everyone else)	0.66	0.34–1.28		
**Protective factors**				
Personal resilience	0.95	0.85–1.06		
Professional fulfillment	0.64**	0.48–0.86	NS	
Leadership support	0.99	0.97–1.01		
**Work risk factors**				
COVID-19 work stressors				
Health worry	1.02	0.94–1.11		
Diagnosis	0.76	0.30–1.89		
Work impact	1.03	0.98–1.08		
Healthcare morally distressing events	1.16	0.91–1.48		
Moral injury				
Self moral injury	3.06**	1.36–6.92	NS	
Others moral injury	1.20	0.70–2.06		
Clinically relevant symptoms (above clinical cutoff)				
Work exhaustion	1.60	0.93–2.76		
Interpersonal disengagement	2.55***	1.48–4.38	1.98*	1.11 to 3.52
Depression	2.53**	1.42–4.50	NS	
Anxiety	4.29***	2.39–7.71	3.83***	2.09 to 7.00
PTSD	4.70***	2.11–10.50	NS	

Multivariable forward conditional logistic regression analyses: Variables with *p* ≤ 0.05 in univariable analyses were included in the multivariable model. Final model *X*^2^(2, *n* = 260) = 30.85, *p* < 0.001. NS, not selected/significant in the final model. *p < 0.05, **p < 0.01, ***p < 0.001.

To address the potential collinearity amongst these predictors and determine which factors were most strongly independently associated with the likelihood of SUC, we entered all significant univariable predictors reported in Table 3 (*p* < 0.05) as potential predictors in a multivariable forward conditional binary logistic regression analysis. The final model, *X^2^* (2, *n* = 260) = 30.85, *p* < 0.001, which correctly classified 70.8% of HCWs, indicated that only two of the six variables, clinically relevant anxiety (OR = 3.83, *p* < 0.001) and clinically relevant interpersonal disengagement (OR = 1.98, *p* = 0.020) were associated with significantly increased odds of SUC. The other variables did not significantly contribute to the prediction and were excluded from the model.

### Variables impacting likelihood of clinically relevant disengagement and anxiety

Because clinically relevant interpersonal disengagement and anxiety were the factors most strongly associated with SUC, we next explored, using post-hoc analyses, which demographic, protective, and risk factors were associated with clinically relevant interpersonal disengagement and anxiety (Tables 4, 5). No demographic factors emerged as significant predictors of interpersonal disengagement, but several protective and risk factors were significantly associated with clinically relevant interpersonal disengagement (Table 3). When these variables were entered together as potential predictors in a multivariable forward conditional logistic regression analysis, three variables emerged as significant predictors. Both COVID-19 work impact (OR = 1.11, *p* < 0.001) and Self MI (OR = 3.51, *p* = 0.010) were significantly associated with increased odds of interpersonal disengagement, while professional fulfillment was associated with decreased odds of interpersonal disengagement (OR = 0.39, *p* < 0.001). The final model, which included all three of these factors, X^2^(3, *n* = 258) = 62.501, *p* < 0.001, correctly classified 74.8% of HCWs. Because professional fulfillment and interpersonal disengagement are derived from the same measure, we used *post hoc* Pearson correlation analyses to explore the overlap between these scales. The correlation between interpersonal disengagement and professional fulfillment was moderate (*r* = −0.58, *p* < 0.001), indicating that professional fulfillment only accounted for 33.9% of the variability in interpersonal disengagement.

**Table 4:** Results of binary logistic regression predicting likelihood of interpersonal disengagement.

**Factors**	**Univariable results**		**Multivariable results**	
	**OR**	**95% CI**	**OR**	**95% CI**
**Demographic factors**				
Age	0.99	0.96–1.01		
Male gender	0.99	0.49–1.98		
White race	1.09	0.58–2.04		
Educational level	0.73	0.49–1.09		
Income	1.03	0.88–1.20		
Married/committeed	0.97	0.57–1.67		
Work in large city	1.08	0.62–1.88		
**Profession**				
Doctor	0.84	0.46–1.52		
Nurse	1.76	0.98–3.17		
Medical assistant	0.85	0.43–1.67		
**Protective factors**				
Personal resilience	0.92	0.82–1.04		
Professional fulfillment	0.36***	0.25–0.51	0.39***	0.27 to 0.56
Leadership support	0.95***	0.93–0.97	NS	
**Work risk factors**				
COVID-19 work stressors				
Health worry	1.05	0.96–1.16		
Diagnosis	2.13	0.90–5.06		
Work impact	1.12***	1.07–1.18	1.11***	1.05 to 1.17
Healthcare morally distressing events	1.51**	1.17–1.94	NS	
Moral injury				
Self moral injury	5.17***	2.21–12.09	3.51**	1.35 to 9.12
Others moral injury	2.08**	1.20–3.60	NS	

Multivariable forward conditional logistic regression analyses: NS, not selected variables for final model, *X*^2^(3, *n* = 258) = 62.50, *p* < 0.001. *p < 0.05, **p < 0.01, ***p < 0.001.

**Table 5:** Univariable and multivariable binary logistic regression predicting likelihood of clinically relevant anxiety.

**Factors**	**Univariable results**		**Multivariable results**	
	**OR**	**95% CI**	**OR**	**95% CI**
**Demographic factors**				
Age	0.96*	0.93–0.99	NS	
Male gender	0.57	0.25–1.30		
White race	0.51*	0.27–0.96	NS	
Educational level	0.56*	0.36–0.88	NS	
Income	0.71***	0.60–0.85	0.74**	0.61 to 0.089
Married/committed	0.48**	0.27–0.85	NS	
Work in large city	1.04	0.57–1.89		
Profession				
Doctor	0.23***	0.10–0.52	NS	
Nurse	0.64	0.88–3.03		
Medical assistant	1.49	0.77–2.91		
**Protective factors**				
Personal resilience	0.82**	0.72–0.93	0.85*	0.74 to 0.98
Professional fulfillment	0.88***	0.84–0.93	0.61**	0.43 to 0.87
Leadership support	0.98	0.95–1.00		
**Work risk factors**				
COVID-19 work stressors				
Health worry	1.14*	1.02–1.26	NS	
Diagnosis	1.59	0.65–3.90		
Work impact	1.03	0.98–1.08		
Healthcare morally distressing events	1.39*	1.08–1.79		
Moral injury				
Self moral injury	4.53***	2.00–10.30	4.00**	1.64 to 9.77
Others moral injury	1.95*	1.09–3.49	NS	

Multivariable forward conditional logistic regression analyses: NS, not selected variables for final model, *X*^2^(4, *n* = 251) = 43.41, *p* < 0.001. *p < 0.05, **p < 0.01, ***p < 0.001.

We next examined which aspects of interpersonal disengagement were most strongly associated with SUC, and which individual items on the COVID-19 work impact and professional fulfilment scales were most associated with interpersonal disengagement, using post-hoc multivariable forward conditional logistic regression analysis. We found that only disengagement from patients was strongly associated with SUC [OR = 1.21, *p* < 0.001; 95% CI 1.09 to 1.33; *X^2^* (1, *n* = 261 = 14.49, *p* < 0.001)], whereas disengagement from colleagues was not a significant predictor in this model.

In the *post hoc* analyses examining the relationships of individual COVID-19 work impact and professional fulfilment items to interpersonal disengagement, no specific work impact items were individually associated, thus suggesting the importance of the cumulative impact of COVID-19. The professional fulfillment items most strongly associated with clinically significant interpersonal disengagement were: “I feel happy at work” (OR = 0.60, *p* = 0.001) and “I feel in control when dealing with difficult problems at work” (OR = 0.61, *p* = 0.002).

As expected, multiple demographic, work, and psychiatric factors were significantly associated with clinically relevant anxiety in univariable analyses (Table 5). When jointly included in multivariable forward conditional logistic regression analyses, only one variable continued to show an association with increased odds of clinically relevant anxiety: Self MI (OR = 4.00, *p* = 0.002). Three variables were associated with significantly decreased odds of clinically relevant anxiety: higher income (OR = 0.74, *p* = 0.002), resilience (OR = 0.85, *p* = 0.027), and professional fulfillment (OR = 0.61, *p* = 0.006). The final model, *X^2^*(4, *n* = 251) = 43.41, *p* < 0.001, correctly classified 75.7% of HCWs.

We again used *post hoc* analyses to determine which individual aspects of resilience and professional fulfilment drove the association with clinically relevant anxiety by including all individual items from each scale in two separate multivariable conditional logistic regressions. The results of the first regression indicated that the resilience item most predictive of clinically significant anxiety was: “Regardless of what happens to me, I believe I can control my reaction to it” (OR = 0.43, *p* < 0.001). The results of the second regression indicated that the professional fulfillment items most predictive of clinically significant anxiety were: “I’m contributing professionally (e.g., patient care, teaching, research, and leadership) in the ways I value most” (OR = 0.66, *p* = 0.003) and “I feel in control when dealing with difficult problems at work” (OR = 0.72, *p* = 0.044).

### *Post hoc* mediation analyses

**Figure 1:**
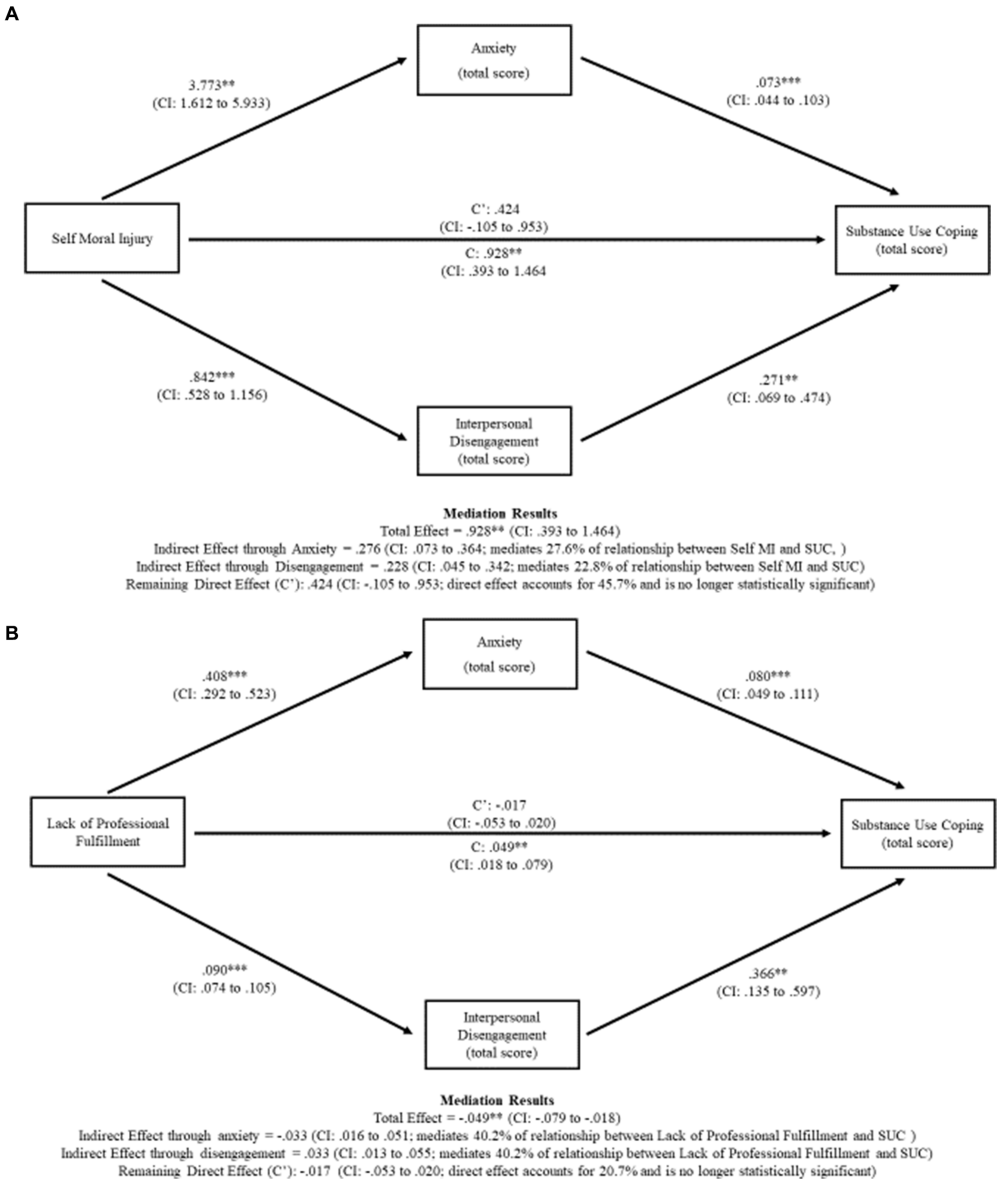
**(A)** Anxiety and interpersonal disengagement mediate the relationship between self-moral injury and substance use coping. **(B)** Anxiety and interpersonal disengagement mediate the relationship between lack of professional fulfillment and substance use coping.

Because Self MI and professional fulfillment were associated with clinically relevant interpersonal disengagement and anxiety, which in turn were associated with increased likelihood of SUC, we next conducted *post hoc* mediation analyses to explore the relationships between these variables. Specifically, we explored whether interpersonal disengagement and/or anxiety mediated the relationship between Self MI and SUC, and between lack of professional fulfillment and SUC. As indicated in Figure 1A, both anxiety and interpersonal disengagement mediated the relationship between Self MI and SUC, *F*(1, 258) = 11.673, *p* = 0.001. Anxiety explained 29.7% of the relationship between Self MI and SUC, whereas interpersonal disengagement explained 24.6% of the relationship between Self MI and SUC. The proportion of total effect of Self MI on SUC operating indirectly through interpersonal disengagement and anxiety was 54.3%; the remaining direct effect of Self MI on SUC was not statistically significant.

Similarly, both anxiety and interpersonal disengagement mediated the relationship between lack of professional fulfillment and SUC, *F*(1, 254) = 9.669, *p* = 0.002 (Figure 1B). Anxiety and interpersonal disengagement each explained 40.2% of this relationship. Thus, the proportion of total effect of lack of professional fulfillment on SUC operating indirectly through interpersonal disengagement and anxiety was 80.4%; the remaining direct effect of lack of professional fulfillment on SUC was not statistically significant.

## Discussion

We found that 35% of HCWs in our study who were surveyed during the COVID-19 pandemic endorsed the use of substances to cope. Even though the overall rates of SUC were relatively low, this finding is nevertheless concerning, as previous work has suggested that HCWs tend to under-report their true rates of SUC (Graham et al., 2001; Weaver et al., 2001; Dumitrascu et al., 2014). As hypothesized, we found that professional fulfillment was associated with lower odds of SUC, while Self MI was associated with higher odds of SUC. Consistent with prior studies (Peterson et al., 2008a,b; Foli et al., 2021a,b), we also found that the likelihood of SUC was higher among HCWs reporting clinically relevant symptoms of depression, anxiety, PTSD, and interpersonal disengagement, although only anxiety and interpersonal disengagement were independently associated with SUC in multivariable analyses.

Although previous studies have linked burnout with SUC (Oreskovich et al., 2015; McCain et al., 2017; Patel et al., 2019), our study found that it was the interpersonal disengagement component of burnout in particular that was associated with an increased likelihood of SUC, while work exhaustion and lack of professional fulfilment were not strongly associated with SUC. This finding is surprising as the rates of work exhaustion (63.1%) were almost twice as high as the rates of interpersonal disengagement (31.6%). We also found that interpersonal disengagement from patients, and not interpersonal disengagement from colleagues, was more strongly associated with SUC. These findings are concerning because disengagement from patients may result in clinical errors that reduce patient care (Kakemam et al., 2021).

We subsequently sought to understand the factors associated with an increased likelihood of interpersonal disengagement and anxiety and found that interpersonal disengagement was not impacted by demographic factors or personal resilience, but was instead associated with work factors. Specifically, the likelihood of interpersonal disengagement was increased in HCWs who experienced Self MI and was also associated with the cumulative impact of caring for COVID-19 patients.

Specifically, we found that demographic characteristics only indirectly impacted SUC coping by impacting the likelihood of clinically relevant anxiety. When variables were entered individually as predictors, we found that individuals who were of younger age, lower income, not in a committed relationship, and not at a doctoral level were more likely to report clinically relevant anxiety. Interestingly, many of these variables relate to the concept of control. Older age, higher income, personal resilience, and professional fulfillment were the protective factors most strongly associated with a decreased likelihood of clinically relevant anxiety. These findings are consistent with prior research suggesting that level of education is negatively correlated to the likelihood of clinically relevant anxiety (Mirza and Jenkins, 2004; Bjelland et al., 2008) and that nurses report greater anxiety than doctors (Hacimusalar et al., 2020).

However, the identified protective demographic factors did not offset the negative impact of Self MI. Our research group has previously reported an association between Self MI and anxiety in this sample, and in this study, we further extend this work to show that Self MI remains a significant predictor of clinically relevant anxiety, even after considering the impact of additional potential risk and protective factors. Consistent with prior research (Kameg et al., 2021), we also found that professional fulfillment was a protective factor for anxiety.

Our results suggest the importance of considering professional fulfillment in the context of SUC among HCWs, as it was associated with decreased likelihood of clinically relevant interpersonal disengagement and anxiety. When examining which aspects of professional fulfillment were most predictive, happiness at work was associated with more interpersonal engagement, contributing professionally was associated with less anxiety, and feeling in control when dealing with work problems was associated with less interpersonal disengagement and anxiety. Similarly, as reported in Dale et al. (2021), we found that it was important to consider the components of MI individually, as Self MI, but not Other MI, was associated with increased likelihood of SUC, although these effects were not direct, as both interpersonal disengagement and anxiety mediated the relationship between Self MI and SUC. These findings provide insights regarding the factors that should be considered in efforts to decrease the rates of substance use coping among HCWs.

We found that the other protective factors of personal resilience and leadership support only indirectly impacted SUC coping by impacting the likelihood of clinically relevant anxiety and interpersonal disengagement. Consistent with prior research (Peñacoba et al., 2021; Setiawati et al., 2021), we found that personal resilience decreased the likelihood of clinically relevant anxiety, which is not surprising as anxiety is likely to be impacted by personal characteristics. When examining which aspects of resilience were most predictive of anxiety, it was the ability to control one’s reactions that was associated with less anxiety. We also found that perceived leadership support decreased the likelihood of interpersonal disengagement, but that it was not a significant predictor after controlling for professional fulfillment. Although we did not explore this further in our analysis, it may be that perceived leadership support impacts the level of professional fulfillment, which then decreases the likelihood of interpersonal disengagement.

### Limitations

While our study reports unique and timely findings, several limitations should be considered. First, the study used a convenience sample, recruited via emails, flyers, and brochures, and we are not able to estimate how many HCWs who viewed the materials chose to not participate or determine the representativeness of the final sample. Our participants come from a specific region of the US and may not be generalizable to other regions in the country. Additionally, the participants were primarily female, and while gender did not significantly predict the outcomes of interest, it may have influenced the findings. Future studies should replicate our findings using different sampling strategies and targeted work settings in order to obtain a more representative sampling of groups of healthcare workers.

The study was cross-sectional and therefore causal assertions cannot be made. Our data included self-report measures that asked about sensitive information, which may have been impacted by respondent biases. Although the study was confidential, respondents may have had a desire to maintain social desirability or avoid any repercussions, which could have impacted their reporting of substance use coping and symptoms of burnout. Future studies should include objective measures of workforce stress such as absenteeism, staff turnover, and disciplinary action.

We used only two questions to assess SUC, both tapping the same underlying construct, which allowed us to binarize participants into those who engaged in SUC and those who did not, but did not allow for a more in-depth assessment of patterns, frequency, and types of SUC. Similarly, we are not able to comment on whether the substance use assessed in our study was indicative of a substance use disorder because we did not evaluate for severity of substance use. Future studies should examine biomarkers of substance use and misuse, along with more specific measures of substance use quantity, frequency and behavioral consequences (i.e., AUDIT, Saunders et al., 1993; or TAPS, McNeely et al., 2016) to get a better understanding of the relationship between risk and protective factors and at-risk substance use among HCWs.

Finally, the healthcare morally distressing experiences we focused on related to quality of care (e.g., not being able to see patients frequently enough) were important but not life threatening. It may be that the inclusion of other patient care experiences (e.g., shortages of ICU beds, triaging of patients to other facilities, and withholding care due to lack of resources) would have produced a more robust measure that would be more linked to the negative outcomes studied, such as interpersonal disengagement and SUC. Future studies should continue to determine which experiences are most morally distressing to HCWs.

### Contributions and implications of study

For patients to receive the highest level of care, it is imperative to ensure that HCWs are functioning well physically and emotionally. It is concerning when providers report using substances for coping, especially as they may be underreporting their use. It is also concerning that SUC was more likely to occur in HCWs experiencing interpersonal disengagement and anxiety, which in turn were associated with their belief that they perpetrated a moral injury and/or were not experiencing professional fulfillment.

It is also striking how much the experience of self MI, although a relatively uncommon occurrence (10% of our sample), increased the likelihood of both interpersonal disengagement and anxiety, even in the context of protective factors such as professional fulfillment. This finding has implications for healthcare systems and supervisors, who should be encouraged to provide support to their employees to decrease moral injury and find ways to increase professional fulfillment.

In particular, HCWs in high risk or high acuity work settings must have support systems in place to prevent interpersonal disengagement, and reduce the risks of SUC. In these settings, it may be important to have systems in place to assess how the HCWs are being impacted by the care they are providing to these patients (Peterson et al., 2008a,b; Davidson et al., 2018). For example, it may be beneficial to use encrypted, anonymous, proactive risk screening to identifying HCWs who are struggling and in need of support.

Interventions targeting these individual and at-risk groups, such as HCWs who are making less income, experiencing moral injury, interpersonal disengagement, and anxiety are also of critical importance. Because mindfulness and meditation have been linked to reduced rates of burnout among HCWs (Goodman and Schorling, 2012; Heeter et al., 2017; Patel et al., 2019), these interventions may be useful in decreasing the levels of anxiety and interpersonal disengagement in HCWs. Although useful, mindfulness strategies are difficult to scale and may not be always be well received by populations that may benefit from them, such as HCWs. Other interventions with possible utility for treating some of the outcomes associated with SUC, such as burnout, moral injury, and anxiety, include eye-movement desensitization and reprocessing (Moench and Billsten, 2021), acceptance and commitment therapy (Otared et al., 2021), app-based technology for monitoring mental health and sleep (Gnanapragasam et al., 2023), and emotional skills training (Ferreres-Galán et al., 2022). It may also be useful to develop prevention strategies that allow HCWs to process the stressors as they are occurring with their colleagues and supervisors. Specifically, they may benefit from peer partnering, distress tracking, psychoeducation, peer support groups, psychological debriefing, and community building activities, which have been proposed as interventions that should be tested high stress work settings (Ellis and Korman, 2022). As suggested by our group and others (Guastello et al., 2022; Meredith et al., 2022), engagement of healthcare leadership in assessing and improving working conditions, and increasing communication and integration across systems can improve both employee engagement and sense of professional fulfillment/accomplishment.

This study shows that HCW sometimes engage in SUC. However, it is not known if this potentially maladaptive substance use results in significant impact on health outcomes. Further research on this and preventive interventions to reduce SUC and potential substance related health consequences is warranted.

## Data availability statement

The raw data supporting the conclusions of this article will be made available by the authors, without undue reservation.

## Ethics statement

The studies involving humans were approved by University of Florida Institutional Review Board. The studies were conducted in accordance with the local legislation and institutional requirements. The participants provided their written informed consent to participate in this study.

## Author contributions

VB, MS, LD, SC, and CM: conceptualization. VB, LD, SC, and CM: methodology. VB, LD, AG, and NS: formal analysis. VB, MS, LD, SC, CM, and BA: writing—original draft preparation. VB, MS, LD, AD, KL, AH, BA, SC, and CM: writing—review and editing. LD and SC: visualization. SC and CM: supervision. CM: project administration. AG: funding acquisition. All authors have read and agreed to the published version of the manuscript.
